# Exploring cyclin-dependent kinase inhibitors: a comprehensive study in search of CDK-6 inhibitors using a pharmacophore modelling and dynamics approach†

**DOI:** 10.1039/d3ra05672d

**Published:** 2023-11-17

**Authors:** Bharath Kumar Chagaleti, Venkatesan Saravanan, Chitra Vellapandian, Muthu K. Kathiravan

**Affiliations:** a Department of Pharmaceutical Chemistry, SRM College of Pharmacy, SRM Institute of Science and Technology Kattankulathur-603203 India kathirak@srmist.edu.in drmkkathir@gmail.com; b Department of Pharmacology, SRM College of Pharmacy SRMIST, Kattankulathur Chennai Tamil Nadu – 603 203 India; c Dr A. P. J. Abdul Kalam Research Lab, Department of Pharmaceutical Chemistry, SRM College of Pharmacy SRMIST, Kattankulathur Chennai Tamil Nadu – 603 203 India

## Abstract

Cancer prevalence and resistance issues in cancer treatment are a significant public health concern globally. Among the existing strategies in cancer therapy, targeting cyclin-dependent kinases (CDKs), especially CDK-6 is found to be one of the most promising targets, as this enzyme plays a pivotal role in cell cycle stages and cell proliferation. Cell proliferation is the characteristic feature of cancer giving rise to solid tumours. Our research focuses on creating novel compounds, specifically, pyrazolopyrimidine fused azetidinones, using a groundbreaking molecular hybridization approach to target CDK-6. Through computational investigations, ligand-based pharmacophore modelling, pharmacokinetic studies (ADMET), molecular docking, and dynamics simulations, we identified 18 promising compounds. The pharmacophore model featured one aromatic hydrophobic centre (F1: Aro/Hyd) and two H-bond acceptors (F2 and F3: Acc). Molecular docking results showed favourable binding energies (−6.5 to −8.0 kcal mol^−1^) and effective hydrogen bonds and hydrophobic interactions. The designed compounds demonstrated good ADMET profiles. Specifically, B6 and B18 showed low energy conformation (−7.8 kcal and −7.6 kcal), providing insights into target inhibition compared to the standard drug Palbociclib. Extensive molecular dynamics simulations confirmed the stability of these derivatives. Throughout the 100 ns simulation, the ligand–protein complexes maintained structural stability, with acceptable RMSD values. These compounds hold promise as potential leads in cancer therapy.

## Abstract 2

Design and development of novel CDK-6 inhibitors by computational investigation using a ligand based pharmacophore model, ADMET studies, molecular docking and dynamics.

## Graphical abstract

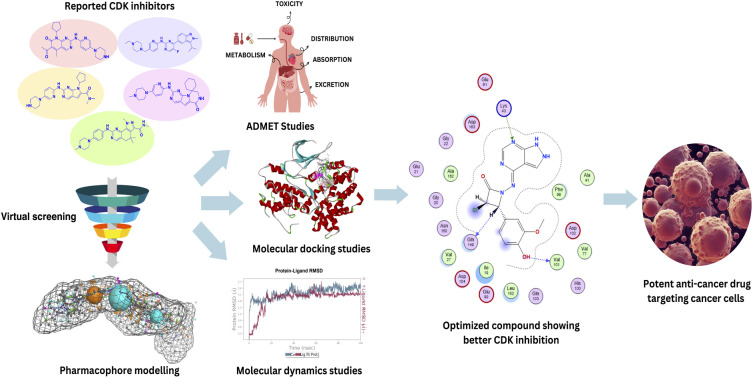

## 1 Introduction

Cancer is the world's second leading cause of death, impacting many individuals globally due to its uncontrolled and continual cell division. According to the American Cancer Society's 2023 projections, the United States alone is anticipated to witness 1 958 310 new instances of cancer and 609 820 cancer-related deaths.^1,2^ Regarding the most commonly diagnosed cancers globally, lung cancer leads with 2.2 million cases, followed by breast cancer (2.09 million), colorectal cancer (1.9 million), prostate cancer (1.28 million), skin cancer (1.04 million), and stomach cancer (1.04 million).^3,4^ Among both genders, the most prevalent malignancies are breast, lung, stomach, colorectal, thyroid, liver, and ovarian cancers. Genetic and epigenetic factors are also responsible for causing the DNA mutation leading to cancer.^5,6^ It is genetically caused by two factors one is the transformation of proto-oncogenes into oncogenes, and another is the deactivation of tumour-suppressing genes. Among the emerging strategies in cancer therapy, targeting cyclin-dependent kinases (CDKs) has gained considerable attention due to the involvement of kinase enzymes in crucial stages of the cell cycle.^7^ In mammalian cells, the regulation of critical cell cycle checkpoints and essential transcriptional processes for cell proliferation is governed by CDKs in response to both external and internal signals.^8^ Catalytic activity is strictly dependent on interaction with cyclinD proteins to play an integral role in cell cycle progression and transcriptional regulation. Numerous CDKs and cyclins have so far been discovered, but particularly CDK–cyclin complexes are essential for regulating cell cycle advancement across the G1, S, G2, and M phases.^9^

Among the various CDK targets from the CDK family, CDK-6 has emerged as a promising target as it is expressed in most common cancers and has a crucial role in driving cell cycle entry and cell progression. Elevated phosphorylation levels of the Rb protein have been observed in various tumours, often linked with the high cyclinD–CDK-4/6 complex expression.^10^ Consequently, focusing on these complexes has emerged as a potential strategy in cancer treatment. Presently, this approach is approved for use in conjunction with the aromatase inhibitor letrozole in the treatment of Estrogen Receptor (ER+) positive, human epidermal growth factor receptor (HER2)-negative breast cancer. Moreover, this therapeutic strategy shows potential for extending its benefits to other solid tumours, such as pancreatic, and biliary cancers, cholangiocarcinoma, and pancreatic ductal adenocarcinoma, offering promising results in combatting various types of cancer.^11,12^ Several particular inhibitors of CDK4 and CDK6 have gained approval from the USFDA for the progressive ER+, HER2-negative breast cancer treatment. Drugs like Palbociclib, Ribociclib, Flavopiridol, and Abemaciclib are a few of the known CDK inhibitors.^13^ Recent studies have underscored the significance of CDK6 in oncogenesis, shedding light on its potential as a therapeutic target. In this context, Researchers are focussing on designing and developing strategies to overcome or prevent drug resistance and improve therapeutic outcomes.

Heterocyclic compounds play a significant role in modern drug discovery and development. Furthermore, researchers have also used various improved synthetic techniques to create these heterocycles, like the molecular hybridization approach. These improved approaches have allowed for the creation of diverse and complex heterocyclic compounds, expanding the possibilities for their applications in designing novel potent hybrids in drug discovery.^14^

**Fig. 1:**
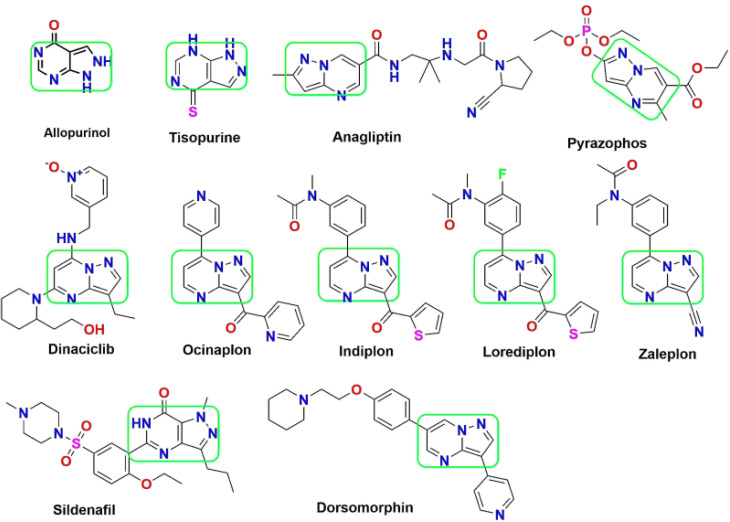
List of pyrazolopyrimidine scaffold as approved drugs including CDK inhibition in cancer therapy.

Among the diverse array of fused heterocyclic motifs, N-fused heterocycles like pyrazolopyrimidine are appealing due to their synthesis feasibility and substantial pharmacological significance on various biological targets. Moreover, these derivatives have emerged as a central focus for researchers aiming to develop inhibitors against these cancer-specific targets. This class of compounds exhibits a wide range of anticancer activity, including cyclin-dependent kinase (CDK) inhibition. Initially recognized as adenosine receptor antagonists, these are implicated in various other roles, acting as CNS depressants, selective COX-1 and COX-2 inhibitors, anti-trypanosomal and sedatives, serotonin 5-HT6 receptor antagonists, corticotrophin-releasing factor (CRF) receptor antagonists, Tuberculostatic agents, and PET tumour imaging agents.^15,16^ Pyrazolopyrimidines have evolved in scientific importance, existing in various isomeric forms such as pyrazolo[3,4-*d*] pyrimidines,^17^ pyrazolo[4,3-*d*] pyrimidines^18–21^ pyrazolo[5,1-*b*] pyrimidines,^22,23^ and pyrazolo[1,5-*a*] pyrimidines.^24–28^ A series of 34 derivatives of 4,6-disubstituted pyrazolo[3,4-*d*] pyrimidines have been synthesized and evaluated for their impact on CDK2/cyclinE kinase. Structure–activity relationship (SAR) studies unveiled that compounds with a thiopentane/thiophenethyl group at C-6 and a heteroatom-containing bicyclic moiety (benzofuran) at C-4 displayed notable CDK2 inhibitory activity.^29,30^ Pyrazolopyrimidines have found in several marketed drugs, including allopurinol, zaleplon, indiplon,^31^ dinaciclib,^32^ dorsomorphin,^33^ ocinaplon,^34^ anagliptin,^35^ lorediplon, pyrazophos,^36^ sildenafil,^37^ tisopurine^38^ and shown in Fig. 1. Derivatives of pyrazolo[3,4-*d*]pyrimidine^39^ exhibit diverse enzyme or kinase inhibitory activities, including targeting Bruton's tyrosine kinase (BTK), cyclin-dependent kinase 7, ataxia-telangiectasia and rad3 related protein (ATR), p70S6K, IL-2 inducible tyrosine kinase, mTOR, glycogen synthase kinase-3β (GSK-3 inhibitors), multiple-mitotic kinase (MMK), PIM-1, Akt/p70S6K, CCchemokine receptor 4 (CCR4), acting as allosteric agonists for the high-affinity nicotinic acid receptor GPR109A, CXCR2 receptor, Src Kinase, mitotic kinesin Eg5, and Janus kinase 3 (JAK3) inhibitors.^40^

**Fig. 2:**
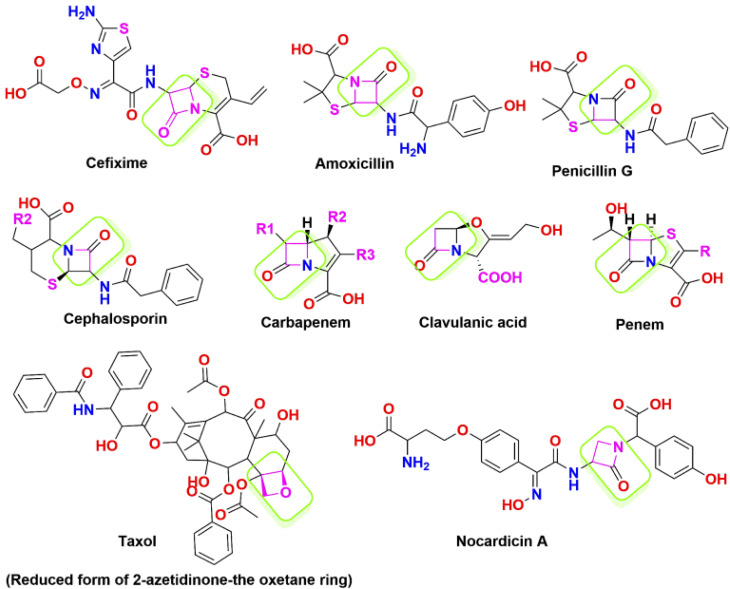
List of marketed drugs containing the azetidinone ring.

The 2-azetidinone structure serves as a distinctive unit and forms the central framework in numerous antimicrobial agents, and mainly presents in penicillin, cephalosporins, nocardicins, and β-lactamase inhibitors.^41,42^Fig. 2 depicts a list of marketed drugs containing the azetidinone ring. Recently, a renewed interest has been in modifying and designing the β-lactam ring to create compounds with a wide range of pharmacological effects. To increase the effectiveness of chemotherapeutic agents in cancer treatment, they additionally serve as pro-drugs to carry the drugs directly to tumour sites. For instance, the semi-synthesis of paclitaxel (Taxol) and docetaxel (Taxotere) has utilized hydroxy lactams with the appropriate substitutions.^43,44^ Furthermore, it has been discovered that β-lactam derivatives cause DNA damage, which causes human leukemic Jurkat T cells to undergo apoptosis.^45,46^

The anti-proliferative activity of 1,4-diarylazetidin-2-ones against the MCF-7 and MDA-MB-231 human breast cancer cell lines has gained remarkable attention.^47,48^ O'Boyle *et al.* have introduced trans derivatives with a phenyl group at the 3^rd^ position, demonstrating an IC_50_ value of 0.8 nm and the ability to inhibit tubulin polymerization in human MCF-7 breast cancer cells.^49^

Consequently, the favourable attributes of these small molecules have been leveraged in developing inhibitors with proposed activity against CDK in the context of this study.

### 1.1 Molecular hybridization approach

A cutting-edge and potent idea in drug development, the molecular hybridization technique uses biologically active compounds to target various diseases successfully.

This method involves the fusion of two parent biologically active molecules (pharmacophore scaffolds), each independently acting on distinct pharmacological targets. The hybrid molecule created by combining two or more pharmacophore scaffolds into a single unit has a more significant pharmacological efficacy than the sum of its component moieties. This innovative strategy involves linking two bioactive scaffolds through a heterocyclic linker, making it an emerging tool in medicinal chemistry and drug discovery.

Two scaffolds with the exact mechanism of action or two drugs with distinctive mechanisms of action can be combined to form molecular hybrids. Compared to the parent drugs, this lessens the chance of drug–drug interactions, minimizes adverse effects, and decreases the tendency to cause drug resistance.^50^ Hybrid anticancer agents have plenty of benefits over standard anticancer medications due to their ability to simultaneously interact with several targets or function on various biological targets. Hybrid anticancer drugs offer remarkable advantages over conventional ones because they are designed to act on different bio targets or interact with multiple targets simultaneously. Properly designed hybrids can effectively target various hallmarks of cancer. They simultaneously prevented the upregulation of resistance mechanisms and circumvented pre-existing resistance in cancer cells. Several methods of molecular hybridization include combining drug pharmacophoric moieties with (a) two pharmacophoric groups directly linked or (b) two pharmacophoric groups linked by a spacer. The development of molecular hybridized scaffolds has significantly accelerated research progress and enabled the design of novel compounds with promising properties and potential therapeutic benefits for cancer treatment.^51^

Computational techniques have long been of value in rational drug design and discovering new hits. Computational resources expedite the drug discovery process, minimizing time and economic expenses. This approach enables the identification of potential drugs with both cost-effectiveness and precise targeting.

**Fig. 3:**
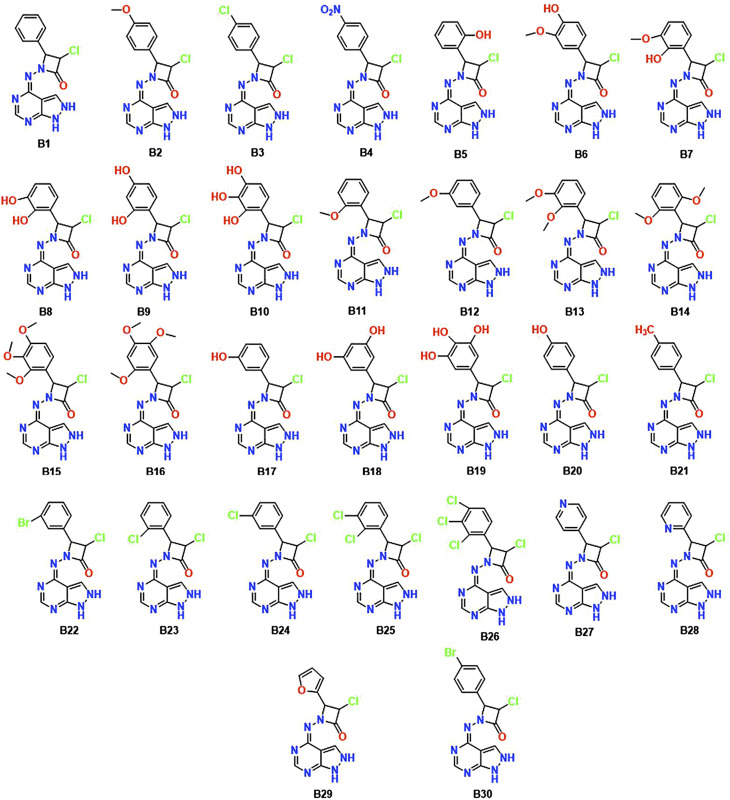
Chemical structures of the newly designed hybrid molecules B1–B30.

Utilizing informatics tools for drug design and virtual screening is important in swiftly acquiring pharmacological products.^52,53^ It is essential to mention that all of the computational methods that are available to identify and evaluate ligand–protein interaction, like pharmacophore modelling, QSAR, molecular docking, dynamics, and ADMET properties had a significant role in drug design and discovery.^54^ Therefore, the main objective of the current research is to create an improved ligand-based pharmacophore model to find novel CDK-6 inhibitors. This work also involves using this methodology to screen a strategically designed database while combining the effective scaffolds of pyrazolopyrimidines and azetidinones. This molecular fusion, achieved using a –NH_2_ linker, falls under the label of a cleavable hybrid. The pyrazolo pyrimidine moiety's –NH_2_ linker demonstrated superior activity, revealed by the SAR studies which tells the association between structure and activity. Based on this context, we designed 30 fused pyrazolopyrimidine with azetidinone through nitrogen linker. Fig. 3 displays the 30 newly created compounds labelled B1 through B30.

The pharmacophoric properties play a significant role in drug development by presenting a set of structural and functional characteristics necessary for a molecule to interact successfully with its target receptor or enzyme. The pharmacokinetic characteristics and potential toxicity of these fascinating hits were computationally analyzed for ADME-T (Absorption, Distribution, Metabolism, Excretion, and Toxicity) studies. Molecular docking and molecular dynamics experiments were also carried out to study the binding affinity and amino acid interactions and to evaluate the molecule stability through RMSF, RMSD, and ligand characteristics.^55,56^

## 2 Rationale and design

The goal of the work is to create a new, potent scaffold that can be utilised to suppress CDK-6 and the associated CDK-6 dysregulation with a synergistic effect by combining pyrazolopyrimidines and azetidinones through a molecular hybridization approach. The designed compounds were subjected to a virtual screening technique known as ligand-based pharmacophore modelling. The initial screening phase involved utilising a previously generated standard ligand-based pharmacophore model from existing drug molecules as a CDK inhibitor. Consequently, pharmacophore mapping was performed to screen the newly suggested structures to identify the hits with the standard features in the designed database toward the target.

**Fig. 4:**
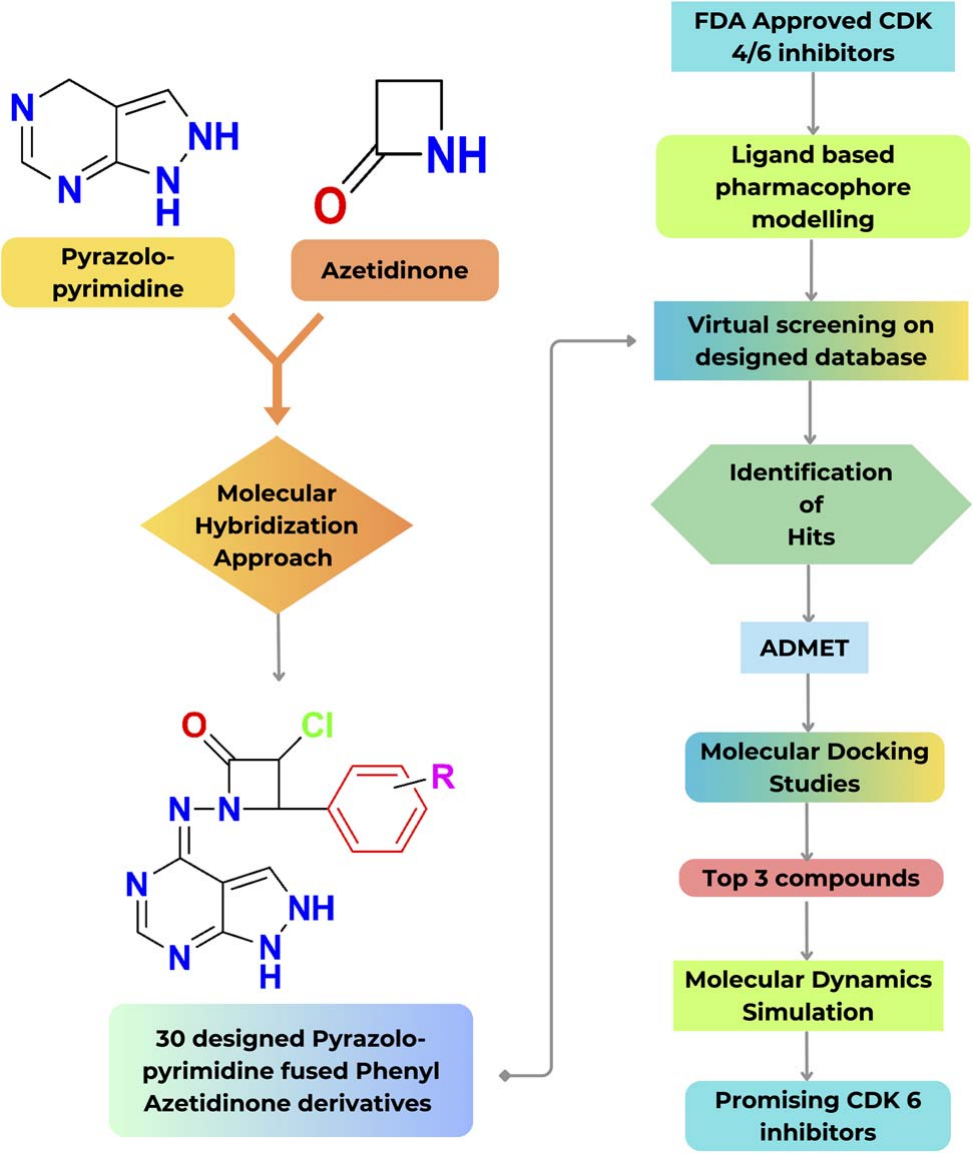
Schematic representation of the present workflow.

Subsequently, pharmacokinetics (ADMET), molecular docking, and simulation studies were performed. A schematic representation illustrating the overall workflow described in the current work is shown in Fig. 4.

## 3 Methodology

### 3.1 Ligand-based pharmacophore model

Pharmacophore modelling is a widely used strategy for finding new hit molecules. Ligand-based drug design relies on the knowledge of known molecules that bind to the target of interest and possess desired biological activity. Understanding these molecules provides valuable insights for designing new compounds and the essential structural characteristics to ensure effective binding to the target molecule.^57^

#### 3.1.1 Training set selection and conformational analysis

In this study, to generate a training pharmacophore model, we initially selected seven standard drugs known for their potent inhibition of cyclin-dependent kinases (Abemacyclib, Palbocyclib, Ribocyclib, Milcyclib, Ecirucyclib, Trilacyclib, and Dinaciclib) and depicted in Fig. S1.† All selected compound's 2D structures were built by using Chem Draw software. These drugs were imported to MOE software and then subjected to energy minimization using the CHARMM-like force field.

#### 3.1.2 Common features of pharmacophore and compounds mapping

Structural information from the training set found a range of features critical for activity, and this data was taken to constitute a pharmacophore hypothesis. To identify the most suitable pharmacophore model, flexible alignment was performed with specific parameters (iteration limit of 100, failure limit of 10, and energy cutoff of 25). Out of 100 conformations, 26 failures and 74 valid conformations were obtained, with a valid score. The best flexible alignment conformation was selected based on the least negative score (S) value and a zero-standard deviation (Sd), resulting in a good conformation model. The Flexible alignment of standard drugs are represented in Fig. S2.†

The newly designed compounds that displayed typical fitting in the pharmacophore model with RMSD values <1 were mapped using the most highly ranked pharmacophore model. RMSD measures the difference between the positions of corresponding atoms in two sets of molecular structures. In the context of pharmacophore modelling, a low RMSD value indicates that the predicted pharmacophore features closely match the actual features of the molecules under study. In essence, a lower RMSD value signifies a higher confidence level in the pharmacophore model's predictive power.^58^

### 3.2 Physicochemical properties and pharmacokinetics studies

Drug likeness analysis is pivotal during drug discovery as it assists medicinal chemists in designing novel compounds. Moreover, a significant contributing factor to the failure of many drugs during clinical trials is their poor ADMET properties and significant toxicity effects on biological systems. The pharmacological effects of a drug are contingent upon its physicochemical attributes, such as the count of rotatable bonds, topological polar surface area, hydrogen bond acceptors and donors, partition coefficient, molecular weight, and various other parameters. The topological polar surface area is a good descriptor characterizing drug absorption, including intestinal absorption, bioavailability, Caco-2 permeability, and blood–brain barrier penetration. Notably, a substantial portion of the marketed compounds adhered to Lipinski's rule of five, which indicates favourable oral bioavailability. Lipinski's rule of five is a valuable tool for assessing essential pharmacokinetic factors encompassing absorption, distribution, metabolism, and excretion (ADME). The investigation followed specific criteria: molecular weight under 500, a maximum of five hydrogen bond donors (HBDs), up to ten hydrogen bond acceptors (HBAs), and no more than five rotatable bonds (RBs). These criteria balance favourable pharmacokinetic properties and the likelihood of successful drug development.^59,60^

This rule proves advantageous in the development of potential therapeutic molecules and the design of drugs. In this experiment, the ADME study was conducted using the SWISS ADME online predictor, a freely accessible tool for evaluating the ADMET properties of small compounds.^61^ Molinspiration, another online tool, unveiled the bioactivity and pharmacokinetic parameters of the provided unidentified compounds. To assess the toxicity of designed compounds, web server vNN-ADMET was utilized.^62^

### 3.3 Molecular docking

Molecular docking has emerged as a valuable method for lead optimization and discovery. Over the past three decades, various docking programs have been developed using multiple search methods and scoring mechanisms. Molecular docking is a computational program identifying possible interactions between the ligand and the target active site.^63,64^ The hit compounds from the Pharmacophore model were then subjected to additional screening utilizing MOE® 2022 molecular docking on CDK6 as a target. The rigid receptor docking method was used to accomplish molecular docking, with the ligands set as flexible and the CDK6 set as rigid. To obtain precise molecular simulations and structural studies within MOE, MMFF94x force field parameters were utilized. From the Protein Data Bank, the X-ray crystal structures of the CDK6 enzyme (PDB ID 2EUF co-crystallized with LQQ, DMS, ACT, and CA ion-free enzyme) were retrieved.^65^ Before starting the docking process, procedure energy minimization was done for the downloaded PDB file. The PDB ID 2EUF was selected for docking since the cocrystal LQQ acted on CDK-6. The selected Protein has two chains, A and B. Protein without water molecules and cocrystals were separated. Subsequently, add polar hydrogens and fix the potential. Further active site prediction by clicking the site finder will give all the active sites present in the target with the size of the active site. Totally 26 active sites are present in the 2EUF protein. The selection of the active site is mainly based on the size of the active site and interactions of the Cocrystal residues.^66–68^

The 30 novel designed ligands were drawn and prepared by MOE, and energy minimization was done. Once the MOE docking protocol's placement scoring and scoring function were combined, the docking procedure was run to simulate the molecular docking of native ligands to the target protein. The MOE® 2022 program was used to visualise and create 3D figures.

### 3.4 Molecular dynamics simulation studies

Molecular dynamics simulation was carried out with Desmond V 5.9 Package Schrodinger LLC suite to analyze the change in the solvent system concerning the macromolecular complex. The docked complex for dynamics was then subjected to an OPLS forcefield.^69^ The position of the complex was centred in an ortho rhombic cubic box, with TIP3P water molecules in addition to the buffers of nearly 10 Å between the protein atom and the box edge for dynamics simulation. The box volume has been computed with complex as well as counter ions like Na^+^ and Cl^−^ ions to neutralize the system.^70^ Under the Desmond protocol, minimization was done using the OPLS-2005 force field parameter, and the Berendsen NVT ensemble was maintained to keep the temperature at 10 K to restrain heavy atoms on the solute. The simulation was performed at a temperature of about 300 K, 1 atmospheric pressure and under a relaxation time of 20 ps.^71,72^ During the simulation process, Martyne–Tobias–Klein barostat and Nose–Hoover thermostat approaches have been utilized for maintaining constant pressure and temperature scale at 1 atm and 300 K, respectively.^73^ The NPT ensemble was initiated, which runs for about 100 ns. The frames have been assembled and scrutinized for investigating the trajectories using simulation interaction diagrams which will be fruitful in determining fluctuations.^74^

## 4 Results and discussion

### 4.1 Pharmacophore model development

The previously described CDK small molecule inhibitors, which displayed a wide range of structural variability and biological activity, were used to construct the 3D pharmacophore model. This study collected a training set of seven reported CDK inhibitors and their chemical structures, shown in Fig. S1.†

**Fig. 5:**
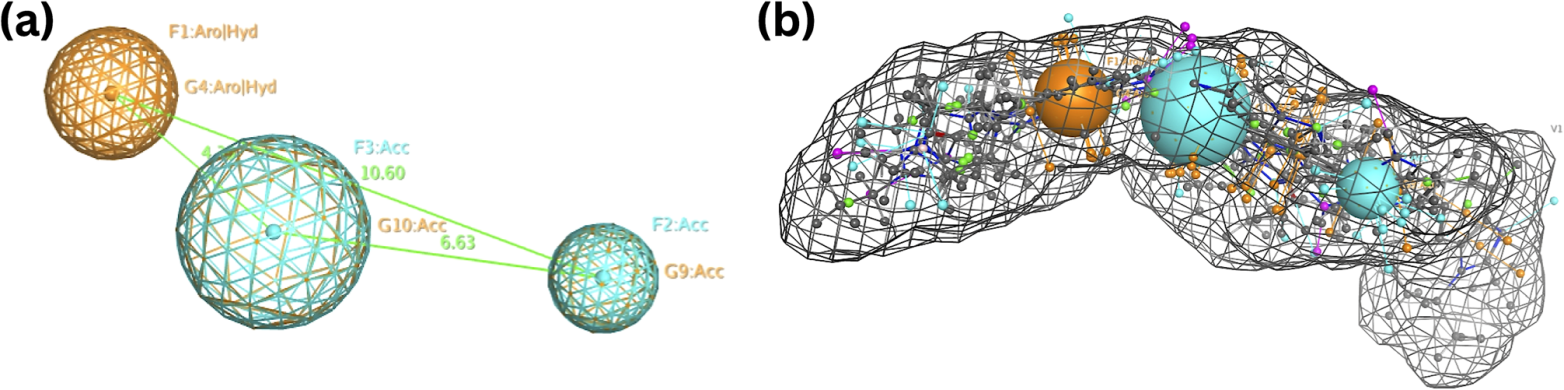
Common features of the developed pharmacophore model generated from the training set alignment of standard drugs.

The best flexible alignment conformation model was utilized to calculate the pharmacophore consensus through the compute-pharmacophore query editor. Initially, a tolerance and threshold of 1.2 and 50% were used, which led to the identification of 12 suggested features denoted as the ph12 training model and saved as ph12.ph4 model. The 12 features are shown in Table 1. To improve the accuracy of the pharmacophore model, we explored various threshold values. When the threshold was set at 60%, 9 features were suggested, denoted as the ph9 training model (saved as ph9.ph4). For a threshold of 80%, 5 features were suggested, designated as the ph5 training model (saved as ph5.ph4). Finally, for thresholds 100%, 3 features were suggested and denoted as the ph3 training model (saved as ph3.ph4). Based on a 100% threshold model, ph3.ph4 was found to be the best pharmacophore model represented by one aromatic hydrophobic centre (F1: Aro/Hyd) and two H-bond acceptors (F2 and F3: Acc) and shown in Fig. 5.

**Table 1:** Pharmacophore consensus

ID	Score	Radius (Å)	Expression
G1	100%	1.10	Aro/Hyd
G2	71%	0.82	Hyd
G3	71%	0.98	Hyd
G4	71%	1.28	Aro/Hyd
G5	71%	1.28	Hyd
G6	57%	1.27	Hyd
G7	57%	1.29	Hyd
G8	100%	1.08	Acc
G9	100%	1.96	Acc
G10	86%	0.93	Acc
G11	86%	1.29	Don
G12	57%	1.12	Acc

#### 4.1.1 Structural consideration to design new CDK inhibitors

Based on the reported literature and molecular hybridization approach, we have designed a series of novel pyrazolopyrimidine-fused azetidinone derivatives targeting cyclin-dependent kinases (CDKs) for anticancer activity.

**Fig. 6:**
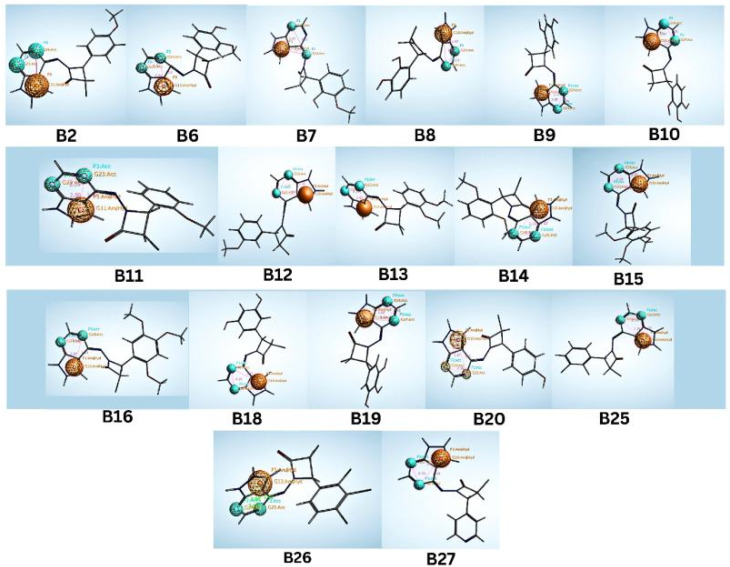
Identified hit molecules from the pharmacophore test set.

These designed compounds were saved as a test data set and subjected to pharmacophore mapping using the ph3.ph4 training model as a standard built with 100% features. This pharmacophore mapping analysis resulted in the identification of 18 hits (B2, B6, B7, B8, B9, B10, B11, B12, B13, B14, B15, B16, B18, B19, B20, B25, B26, and B27) out of the 30 test compounds, exhibiting an impressive 100% match with the pharmacophoric features and RMSD values are less than 1. Moreover, the RMSD value is less than one, suggesting that the predicted pharmacophore is in good agreement with the experimental data, meaning that the model is reliable and can accurately represent the essential structural and functional elements responsible for the biological activity of the compounds. The RMSD results of pharmacophore hits are shown in Table 2 and Fig. 6.

**Table 2:** Pharmacophore hits RMSD

S. no.	Ligand i.d.	Pharmacophore RMSD (Å)
1	B2	0.44
2	B6	0.43
3	B7	0.61
4	B8	0.74
5	B9	0.43
6	B10	0.47
7	B11	0.92
8	B12	0.77
9	B13	0.68
10	B14	0.84
11	B15	0.65
12	B16	0.23
13	B18	0.76
14	B19	0.50
15	B20	0.53
16	B25	0.93
17	B26	0.93
18	B27	0.93

#### 4.1.2 Pharmacophore mapping

With RMSD values ranging below one, the data indicated a significant affinity between the 18 compounds evaluated within the selected pharmacophore model. This RMSD of pharmacophore results being less than one is to ensure a high level of accuracy, and all these compounds retained the fundamental structural characteristics outlined in the pharmacophore model, as visually represented in Fig. 6.

Notably, it was found that compounds fitted the model where the two H-bond acceptors (F2 and F3: Acc) were represented by the pyrimidine and azetidinone rings and the one aromatic hydrophobic centre (F1: Aro/Hyd) centre by the phenyl or pyrazole ring moieties. While aromatic hydrophobic interactions are essential for anchoring a molecule within the hydrophobic binding sites of a target protein and can help increase the stability of the drug–receptor complex, pharmacophoric structural characteristics have more significance. Aromatic interactions are useful for controlling the activity of a target protein since they are frequently involved in important biological processes. Similar to hydrogen bond donors (such as amine or hydroxyl groups), hydrogen bond acceptors can also create hydrogen bonds with them. Given that there are two hydrogen bond acceptors, the molecule can likely make vital hydrogen bonding connections with the complementary donor groups on the target protein. Due to these interactions, the ligand and CDK target may benefit from favourable binding and structural complementarity, increasing total binding affinity.

### 4.2 Physicochemical properties

Physicochemical parameters of the designed compounds were predicted using Swiss ADME software, and all the results are shown in Table S1.† Most “drug-like” compounds have log *P* = 5, a molecular weight = 500, several hydrogen bond acceptors = 10, and several hydrogen bond donors = 5, in accordance with the Lipinski rule. The bioavailability of molecules that violate more than one of these principles may need to be revised. log *P* values for all molecules were in the range of 0.25 to 3.43 Å. This shows good oral bioavailability of the compounds.

Furthermore, all compound's molecular weight should be less than 500 Daltons, and all the compounds were less than 500, between 304.70–418.07. Hydrogen bond acceptors and donors are within the range. Accordingly, TPSA should fall under 140 Å, indicating that the compound has good penetration properties. All the compounds fall under these criteria, except for compounds B10 and B18, where the value was above 150 Å. The number of rotatable bonds is between 2 to 5 within the range, suggesting that the molecules are highly flexible. These rotatable bonds also influence their pharmacokinetics, binding interactions, metabolism, and overall efficacy. The compounds did not depict any violations, and all the compounds followed Lipinski's rule of five.

#### 4.2.1 Bioactivity studies

The bioactivity score is the calculated value that represents the predicted biological activity of a chemical compound. It is a numerical estimation of the compound's potential to exhibit specific bioactivity, such as enzyme inhibition, receptor binding, or other biological effects. Having a high score is considered to illustrate good activity. So, having a score above 0.00 is considered a molecule with good bioactivity, and if the scores are between −0.50 to 0.00, they are considered moderately active compounds. The compound is considered inactive if the value is below or less than −0.50. The bioactivity score of the designed compounds was predicted using molinspiration software, and all the results are shown in Table S2.†

The designed compounds results showed moderate activity for GPCR ligand and in between the range of −0.50 to −0.31. For the ion channel target, results were in the range of −0.30 to −0.49, and compound B29 depicted inactive results as it was above −0.50. Interestingly for the kinase inhibitor, the values were obtained positive and between 0.13 to −0.33. Compounds B1, B3, B5, B6, B8, B9, B10, B17, B18, B19, B20, B27, B28 showed good activity. The compounds were inactive for Nuclear Receptor Ligand and Protease inhibitors as they were above the range of −0.50. Likewise, the Enzyme Inhibitor activity was moderate, between −0.20 to −0.49. The only kinase inhibitor demonstrated positive results when compared to the other targets.

#### 4.2.2 ADMET studies

Table S3† illustrates the ADMET studies of all designed compounds. Although none exhibit cytotoxicity, all compounds exhibit drug-induced liver injury. Likewise, for metabolism studies, only compound B12 did not show any such property, while the remaining compounds had shown. Furthermore, none of the compounds had inhibitory action against cytochrome 1A2, 3A4, 2D6, 2E9, and 2C19. All compounds B4, B7, B9, B13, B14, B15, and B20 cannot cross the blood–brain barrier (BBB), while the remaining compounds exhibit BBB permeability. For PgP Inhibitors, compounds B2, B6, B11, B12, B13, B15, B21, B22, B23, B24, B25, B26, and B30 showed the properties of the same, while the remaining compounds did not. Likewise for substrate activity B2, B7, B11, B12, B13, B14, B15, B16 illustrated the activity for the same. HerG blocker activity for cardiotoxicity was shown by compounds B1, B3, B11, B12, B14, B16, B17, B22, B23, B24, B27, B28 and B29.

### 4.3 Molecular docking

The docking score results of the designed (B1–B30) series were mentioned in Table 3, giving an idea about the affinities of the ligand with the receptor. The designed compounds were compared with the standard Palbociclib. Each compound underwent five conformations and was given an accurate binding score. Among the designed title series, B6 and B18 showed more potent towards the target and exhibited a good binding affinity of −7.8 kcal^−1^ and −7.6 kcal^−1^ compared to the Standard drug Palbociclib. The binding Energy of the standard drug Palbociclib is −8.0 kcal^−1^, and the interactions are Val101, Asp163, (Hydrophobic) Leu152 (H-bond). The interactions of compound B6 are Val101, Gln149, (hydrophobic) Lys43, (H-bond) and B18 are Val101, Asp163, (H-bond) Gly20, Asp104 (hydrophobic). The compounds B6, B18 and standard interacted similarly with the amino acid Val 101.

**Table 3:** CDK6-ligand interactions recorded during docking

Ligand i.d.	Docking score (kcal mol^−1^)	Number of interacting residues	Amino acid interaction
B1	−6.7	1	Lys 43, (H-bond)
B2	−7.3	2	Lys43, Glu61, (H-bond)
B3	−7.1	2	Val101, (H-bond), Glu21, (hydrophobic)
B4	−7.5	6	Lys43, Glu61, Lys147, Asn150, Gln149, (H-bond), Gly22, (hydrophobic)
B5	−6.9	5	Lys43, Asp163, Phe98, (H-bond), Gly20, Val 101, (hydrophobic)
B6	−7.8	3	Val101, Gln149, (hydrophobic), Lys43, (H-bond)
B7	−7.2	2	Lys43, (H-bond), Gln49, (hydrophobic)
B8	−6.6	2	Asn150, (H-bond), Val101, (hydrophobic)
B9	−7.1	4	Lys43, Phe98, Val27, (H-bond), Val101, (hydrophobic)
B10	−7.0	2	Val101, Glu21, (hydrophobic)
B11	−7.2	0	—
B12	−7.4	0	—
B13	−7.3	3	Lys43, Asp163, (H-bond)
B14	−7.1	1	Lys43, (H-bond)
B15	−7.5	2	Lys43, (H-bond)
B16	−7.3	3	Ile19, Thr107, Gln149, (H-bond)
B17	−6.7	2	Asn150, (H-bond), Val 101, (hydrophobic)
B18	−7.6	4	Val101, Asp163, (H-bond), Gly20, Asp104, (hydrophobic)
B19	−7.0	4	Val101, Asp104, (H-bond), Asp163, Gly20, (hydrophobic)
B20	−7.0	2	Val101, Glu21, (hydrophobic)
B21	−7.1	2	Lys43, Glu61, (H-bond)
B22	−7.2	1	Lys43, (H-bond)
B23	−6.8	1	Lys43, (H-bond)
B24	−6.8	3	Lys43, Glu61, Phe98, (H-bond)
B25	−7.1	1	Lys43, (H-bond)
B26	−7.2	1	Lys43, (H-bond), Gln149, (hydrophobic)
B27	−6.9	2	Lys43, Glu61, (H-bond)
B28	−6.9	2	Lys43, Glu61, (H-bond)
B29	−6.5	3	Lys43, Glu61, Phe98, (H-bond)
B30	−7.2	2	Lys43, Glu61, (H-bond)
Palbociclib	−8.0	3	Val101, Asp163, (hydrophobic) Leu152, (H-bond)

**Fig. 7:**
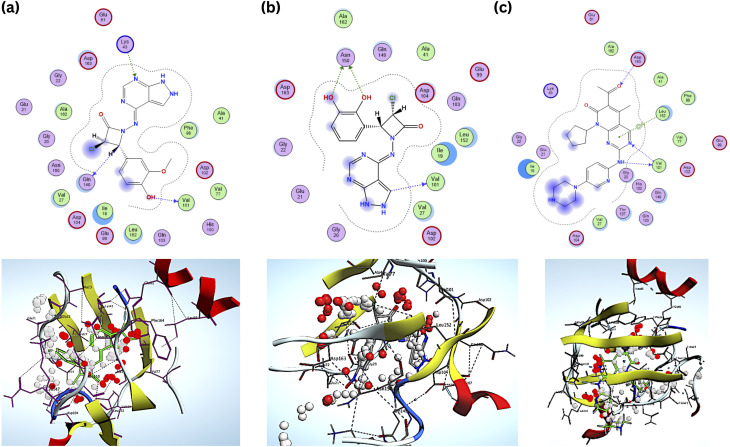
2D & 3D interactions of the (a) compound B6 (b) B18 and (c) standard.

**Fig. 8:**
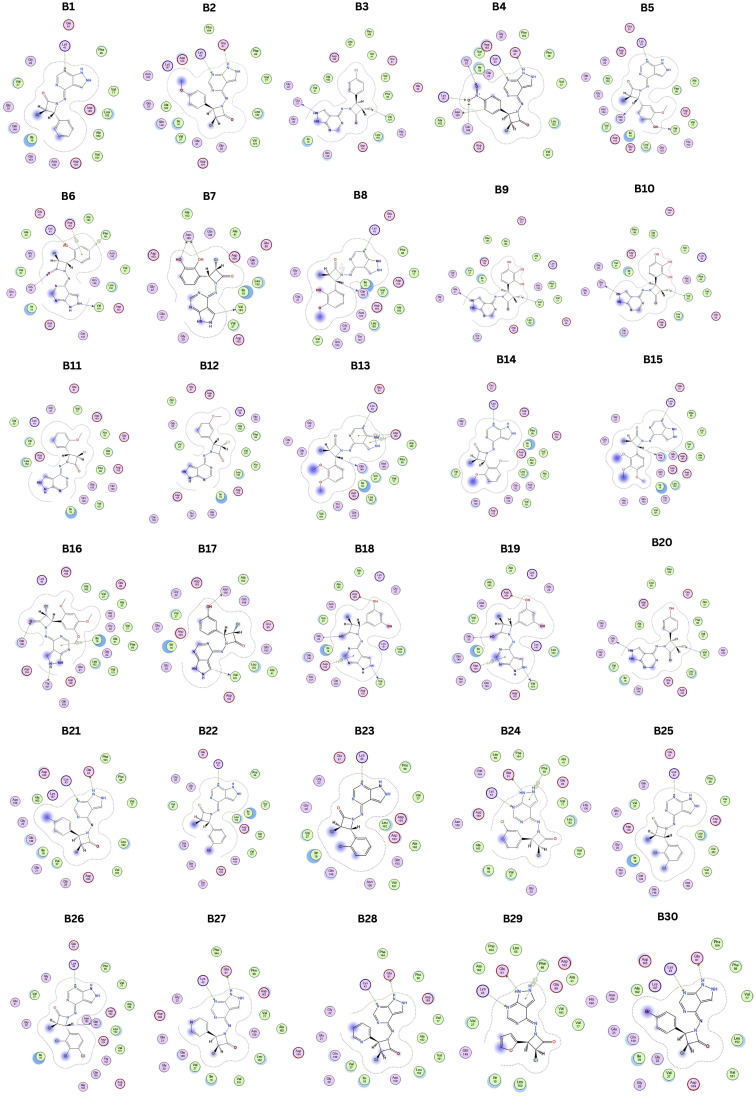
2D interactions of designed hybrid molecules.

Compared to the standard, compound B18 interactions are interlinking with the same amino acids, *i.e.*, Asp163, Val 101. The 2D & 3D interactions of the Compounds B6 and B18 at the active site of the 2EUF are depicted in Fig. 7. Based on this obtained similar interactions and better binding scores, these compounds were further subjected to molecular dynamics studies to know the stability of the compound with the protein. Other compounds also showed near and better significant binding affinity when compared with the standard. 2D interactions of all compounds are shown in Fig. 8.

### 4.4 Molecular dynamics

#### 4.4.1 RMSD

**Fig. 9:**
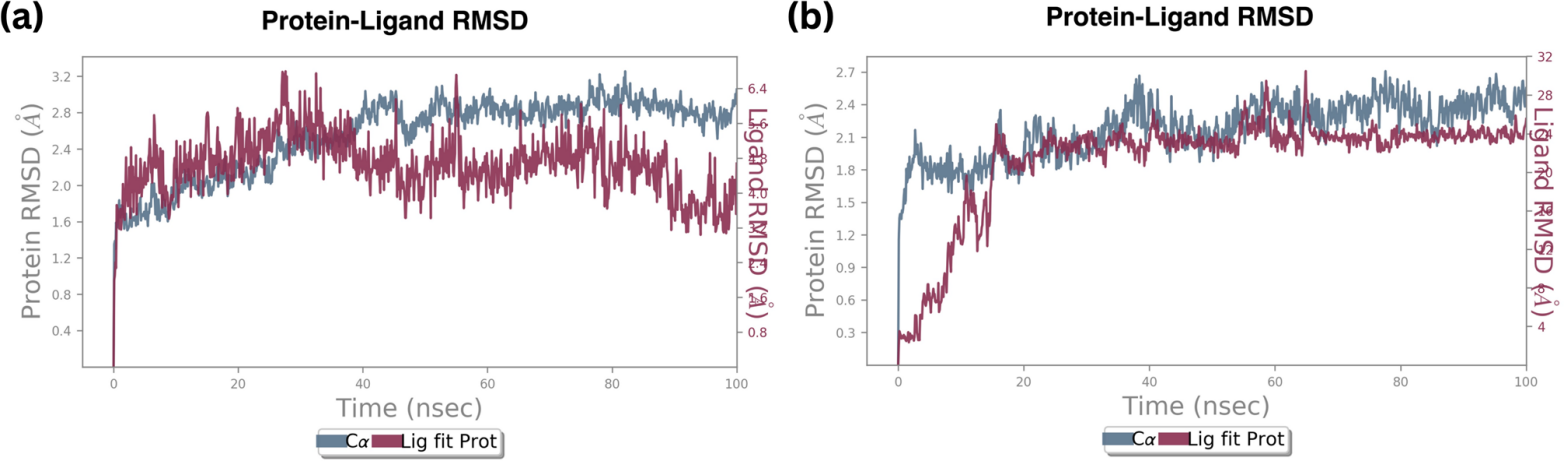
RMSD plot of ligand fit protein of (a) standard Palbociclib, (b) compound 6.

The Root Mean Square Deviation (RMSD) is a key parameter indicating the stability and fluctuations of a protein–ligand complex during the simulation. The mobility of the loops can be used to explain the significant RMSD value. A visual trajectory study also confirms the stability of the protein's secondary structure. Based on the simulation results, the backbone structure and C alpha residues were examined. The ligand fit protein of compound B6 demonstrated positive and negative variations for 15 nanoseconds (0.3–2.1 Å) before achieving stability at 2.1 and remaining stable throughout the simulation. The RMSD average value of the protein backbone is 2.2 Å. For the standard (Palbociclib) molecule complex, the average and maximum values were 2.4 and 3.2 Å at 30 nanoseconds, as shown in Fig. 9a.

In contrast, the RMSD of a protein and compound B6 complex backbone began at 0.3 Å at 0.1 nanoseconds, then attained maximum and minimum values at 2.4 Å and 2.1 Å, respectively. At the same trajectory, it stabilized at 15 ns of the simulation time, and the stability was maintained till the end of the simulation, as shown in Fig. 9b Compared to standard molecule complexes, these values showed higher structural refinement throughout the investigation, indicating that the system has equilibrated. The simulation will be long enough for rigorous analysis. The RMSD of a protein and compound 18 complexes in Fig. S3† indicates the backbone was initially started at 0.9 Å. The average value of the protein backbone is 1.8 Å and achieved maximum at 2.4 Å at specific trajectories but attained stability around 2.1 Å at 20 ns and remained stable till 50 ns of the simulation.

The average value of the complex is high, about 0.2 Å when compared with complex B6. From these findings, the compound B18 complex has deviated from compound B6. However, they are mutually compared with their RMSD values and complex values. It signifies that Cα, the protein backbone is well correlated. Still, the ligand fit protein fluctuated around 0.1 nanoseconds at 0.9–1.5 Å, then reached medially around 2.0 Å at 20 nanoseconds and stability was maintained till 50 ns then continuously fluctuated till 100 nanoseconds from 0.5–2.5 Å.

Root mean square deviation (RMSD) values of the Cα atoms of the protein attained the stable state within 2.0 to 3.0 deviations from its initial structure, according to the analysis of the produced trajectories of the complexes. This states that upon binding of compound B6, the protein retains the interaction profile and does not suffer further significant conformational changes. Between compound B6 and compound B18, compound B6 was the most stable based on the RMSD value. However, compound B6 demonstrated remarkable stability, maintaining a consistent fluctuation throughout the simulation. This steadiness suggests a strong and stable interaction between B6 and the protein, indicating a potential robust binding affinity over time.

#### 4.4.2 RMSF

When a ligand binds to a protein, it may cause structural alterations in the protein that may alter the RMSF plot. The ligand's interaction is likely causing the protein structures' stability to break down, which would increase fluctuations in the RMSF plot. Changes in hydrogen bonds, van der Waals interactions, or electrostatic interactions between the protein and the ligand could all be contributing factors. As the protein–ligand complexes have lesser RMSF values than the apoprotein, this may indicate that the protein structure is stabilized because of the ligands binding. Moreover, the binding of a ligand might make the protein structure more flexible overall, which would cause the RMSF values to fluctuate more, which is not necessarily indicative of instability. Ligand binding can induce changes in the protein structure that allow it to adopt multiple conformations, leading to increased flexibility.

The RMSF graph shows that there are no local changes along the protein chain during thermal motion. Mild helical fluctuations and moderate loop fluctuations can be observed. Strong hydrogen bond interactions are indicated by vibrations greater than 4.5. The ‘N’ and ‘C’ end terminals of the protein changed more than in other areas. The secondary structure components with more rigidity, such as -helices and -strands, fluctuated less than loop region residues, which took part in 70% of the simulation period. The protein backbone has an average RMSF value of 3.0. As usual, the protein RMSF value of compound B6 complex indicates that the ‘N’-terminal and ‘C’- end terminal fluctuate more than α-helices and β-strands. In the RMSF graph, the most important backbone residue positions are LEU 8 with 0.4 fraction, which means 40% of the simulation time, the specific interaction is maintained, GLU 67 with 0.5 fraction, ASP 69 with 0.35 fraction, and GLN 73 with 0.3 fraction. The protein RMSF B6 complex indicates more fluctuations in the ‘N’ and ‘C’ end terminals.

Moreover, loop regions fluctuated at a higher range showing ‘H’ bonding interactions. Compared with compound B6 complex and standard molecule, the compound B6 complex, RMSF fluctuations were higher and above 5.0 Å in its unstructured portion of the protein. These values are much better when compared with standard molecules and deviate from the limits of compound B6 complexes. Further, the minimum value of the compound B6 complex positively contributes to the stability during simulation showing amino acid interactions at different trajectories. The protein RMSF and ligand RMSF of compound B6 and standard are shown in Fig. S4.†

#### 4.4.3 H bond analysis

**Fig. 10:**
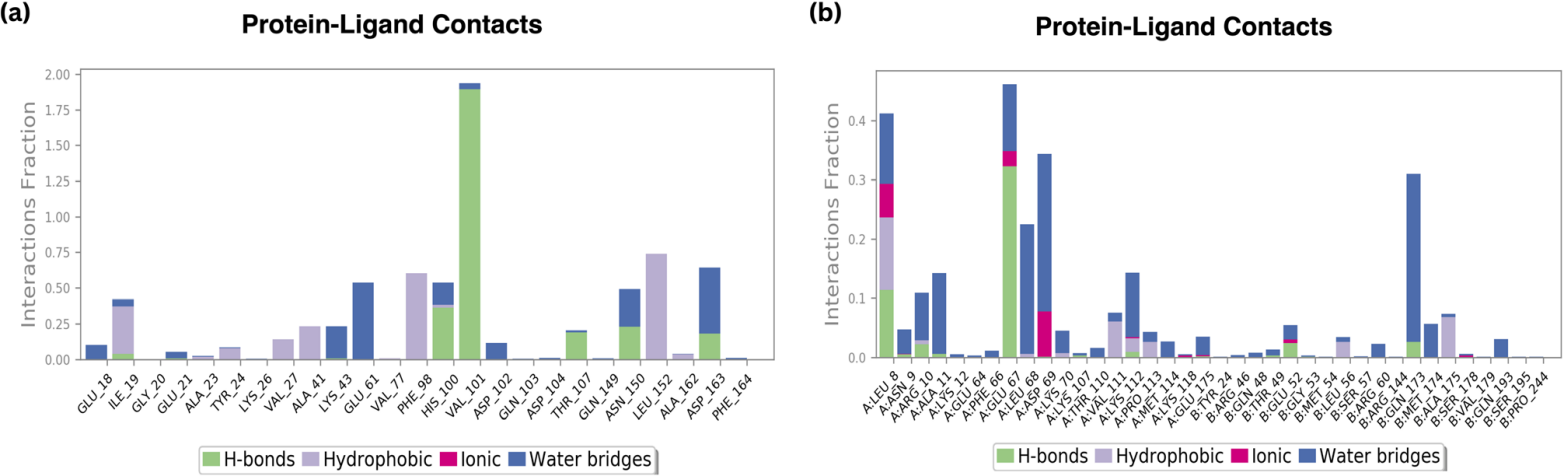
H-bond plot of (a) standard Palbociclib (b) compound 6.

A histogram graphic displaying the protein–ligand contacts was generated using the dynamics calculations as a validation parameter to the docking results to determine the significant interacting amino acids contributing to biological activity. The complex arrangement of ‘H’ bonds, hydrophobic contacts, water bridges, and ionic interactions. Fig. 10a illustrates the interactions at the maximum of 80% of the simulation time for the standard compound at residues HIS 100, VAL 101, and ASP 163 and Fig. 10b a shows the interactions of three H bonds at residues LEU 8, GLU 67, and GLN 173 at a maximum of 40% of the simulation time for compound B6, Fig. S3† depicts the interactions at a maximum of 60% of the simulation time for compound 18.

According to the findings, all three compounds maintained their docking connections by forming hydrogen bonds, hydrophobic, water bridges, and salt bridges.

#### 4.4.4 Secondary structure elements

The preservation of secondary structure elements is crucial for the protein's biological function. Changes in secondary structure, especially in key regions, can influence the protein's binding affinity, stability, and overall activity. The secondary structure elements, particularly α-helices and β-strands, play a critical role in determining the overall stability and function of proteins. In the standard drug simulation, the protein exhibited a notable presence of α-helices, accounting for 35.68% of the protein's secondary structure, while β-strands constituted 7.79%. Together, these elements comprised 43.47% of the total secondary structure elements throughout the simulation period.

Interestingly, in the presence of compound B6, the protein maintained a similar structural profile. The percentage of α-helices slightly decreased to 35.23%, while β-strands increased marginally to 8.41%. Consequently, the total secondary structure elements constituted 43.64% of the protein's structure during the simulation.

The comparable secondary structure profiles between the standard drug and compound B6 indicate that B6 does not significantly disrupt the native protein structure. This is a positive finding, suggesting that B6 interacts with the protein without inducing major conformational changes. The subtle variations observed in the α-helix and β-strand percentages could be attributed to the specific interactions between the compound and the protein residues, possibly stabilizing certain secondary structure elements. The fact that both the standard drug and compound B6 maintain similar secondary structure profiles implies that B6 may have a similar or potentially enhanced therapeutic effect compared to the standard drug.

#### 4.4.5 Ligand properties

##### 4.4.5.1 Radius of gyration

The radius of gyration provides insights into the compactness of the protein–ligand complex. A smaller radius indicates a more compact and stable structure. In this study, the radius of gyration for compound B6 was consistently less than that of the standard compound. This implies that the protein-ligand complex formed by B6 is more compact and structurally stable compared to the complex with the standard compound. The compact nature of the complex is often indicative of a strong and specific binding interaction.

##### 4.4.5.2 Molecular surface area

The molecular surface area provides information about the exposed surface of the protein–ligand complex. In this study, the standard compound exhibited a larger molecular surface area (414 Å^2^) compared to compound B6 (310 Å^2^). While a larger surface area can imply a broader interaction interface, it doesn't necessarily correlate with stronger binding. The slightly smaller surface area of the B6 complex suggests a more specific and targeted binding mode, which can be advantageous in drug design by minimizing off-target interactions and potential side effects.

#### 4.4.6 Protein–ligand contacts

##### 4.4.6.1 Amino acid interactions

B6 displayed favourable amino acid interactions, notably with GLU67 and LEU8. These interactions not only involve hydrogen bonds but also incorporate hydrophobic contacts and ionic interactions, contributing significantly to the ligand's stability within the binding site. The balanced combination of these interactions is indicative of B6's potential as an effective therapeutic agent.

Conversely, the standard drug exhibited interactions with HIS100 and VAL101, which are crucial for its stability. However, the presence of moderate interactions with THR107, ASN150, and ASP163 suggests opportunities for further improvement in its binding profile.

##### 4.4.6.2 Hydrophobic and ionic interactions

Both B6 and the standard compound maintained comparable hydrophobic contacts. However, B6 showcased a distinct advantage in achieving favourable amino acid interactions, particularly with GLU67 and LEU8. These interactions, along with the observed ionic interactions, contribute significantly to the ligand's stability, possibly enhancing its efficacy and specificity in inhibiting the target protein.

#### 4.4.7 Solvent accessible surface area (SASA) analysis

The Solvent Accessible Surface Area (SASA) analysis provides valuable insights into the interaction of molecular surfaces with solvent molecules, indicating the accessibility and exposure of the molecules to the surrounding environment during the simulation. In the case of compound B6, the SASA values exhibited fluctuations ranging from approximately 220 Å to 500 Å. Notably, B6 maintained a relatively constant SASA value, indicating consistent exposure to solvent molecules throughout the simulation period. This stability suggests that B6 sustains interactions with the surrounding solvent, potentially facilitating its role in biological processes. The ability of B6 to maintain a stable SASA suggests its resilience in various solvent environments, a crucial factor for its effectiveness as a therapeutic agent.

Conversely, the standard compound displayed fluctuations in SASA values ranging from about 150 Å to 240 Å. Unlike B6, the standard compound exhibited continuous fluctuations throughout the simulation time, suggesting varying degrees of exposure to solvent molecules. This dynamic behaviour could imply potential challenges in maintaining stable interactions with the surrounding environment. The continuous fluctuation in SASA values for the standard compound might impact its stability and could have implications for its efficacy in practical applications. B6's ability to maintain a more stable SASA profile suggests a robust and consistent interaction with solvent molecules, indicating its potential suitability for various biological environments. On the other hand, the standard compound's continuous SASA fluctuations might raise concerns about its stability and long-term effectiveness.

## 5 Conclusion

In summary, our research signifies a significant advancement in cancer treatment, demonstrating the potential of pyrazolopyrimidine-fused azetidinone hybrids as promising lead molecules. To decipher CDK6's mechanism of action, we generated common feature pharmacophore models. Through thorough pharmacophore modelling and simulation studies, we unveiled the crucial role of aromatic hydrophobic centres and hydrogen bond acceptor features in designing potent compounds. The pharmacophore model featured one aromatic hydrophobic centre (F1: Aro/Hyd) and two H-bond acceptors (F2 and F3: Acc). These identified structural features play a pivotal role in anchoring ligands within CDK6's hydrophobic binding pockets, enhancing the drug–receptor complex stability. Additionally, designed derivatives B1–B30 have a good ADMET (absorption, distribution, metabolism, excretion, toxicity) profile. Our molecular docking results highlighted compounds B6 and B18 as potent CDK6 binders. B6, exhibiting a low energy conformation of −7.8 kcal mol^−1^ with OH at the *para* position and OCH_3_ at the *meta* position substitutions, displayed superior stability in the CDK6 complex compared to the standard drug Palbociclib. Compound B6 maintains a stable interaction with the CDK6, characterized by its low and consistent RMSD values, smaller radius of gyration, and absence of intramolecular hydrogen bonds. Additionally, compound B6 establishes stable hydrogen bonds, hydrophobic contacts, and ionic interactions, particularly with GLU67 and LEU8 residues. These results underscore the compact and stable nature of the B6–CDK6 complex, emphasizing its potential as a promising therapeutic candidate for cancer treatment. The findings from the current study support the notion that the tested compounds are effective CDK-6 inhibitors for cancer treatment. Furthermore, these compounds may serve as promising starting points to expedite drug discovery and development against the cyclin-dependent kinase-6 domain in future scenarios. These discoveries open new avenues for innovative and targeted cancer therapies, bringing us closer to more effective treatments and improved outcomes for patients battling this devastating disease.

## Author contributions

BK: data collection, conceptualization, data curation, investigation, methodology, validation, writing-original draft. VS: writing & editing, CV: resources, software, supervision, MK: resources, software, supervision, writing-review & editing.

## Conflicts of interest

There are no conflicts to declare.

## Supplementary Material

RA-013-D3RA05672D-s001
