# Supplementary material for: Exploring cyclin-dependent kinase inhibitors: a comprehensive study in search of CDK-6 inhibitors using a pharmacophore modelling and dynamics approach
Source: RSC Adv. 2023 Nov 17;13(48):33770–85. doi: 10.1039/d3ra05672d (PMC10655667; doi:10.1039/d3ra05672d)
Supplement: RA-013-D3RA05672D-s001 [file RA-013-D3RA05672D-s001.pdf]

Electronic Supplementary Material (ESI) for RSC Advances.
This journal is © The Royal Society of Chemistry 2023

Exploring Cyclin-Dependent Kinase Inhibitors: A Comprehensive Study in Search of CDK-6 Inhibitors using Pharmacophore Modelling and Dynamic Approach

Bharath Kumar Chagaleti,[a] Venkatesan Saravanan,[a] Chitra Vellapandian,[b] and Muthu K Kathiravan*[a,c]

[a.] *Department of Pharmaceutical Chemistry, SRM College of Pharmacy, SRM Institute of Science and Technology, Kattankulathur-603203, India.*
[b.] *Department of Pharmacology, SRM College of Pharmacy, SRMIST, Kattankulathur, Chennai, Tamil Nadu – 603 203, India*
[c.] *Dr. A. P. J. Abdul Kalam Research Lab, Department of Pharmaceutical Chemistry, SRM College of Pharmacy, SRMIST, Kattankulathur, Chennai, Tamil Nadu – 603 203, India. E-mail:drmkkathir@gmail.com*

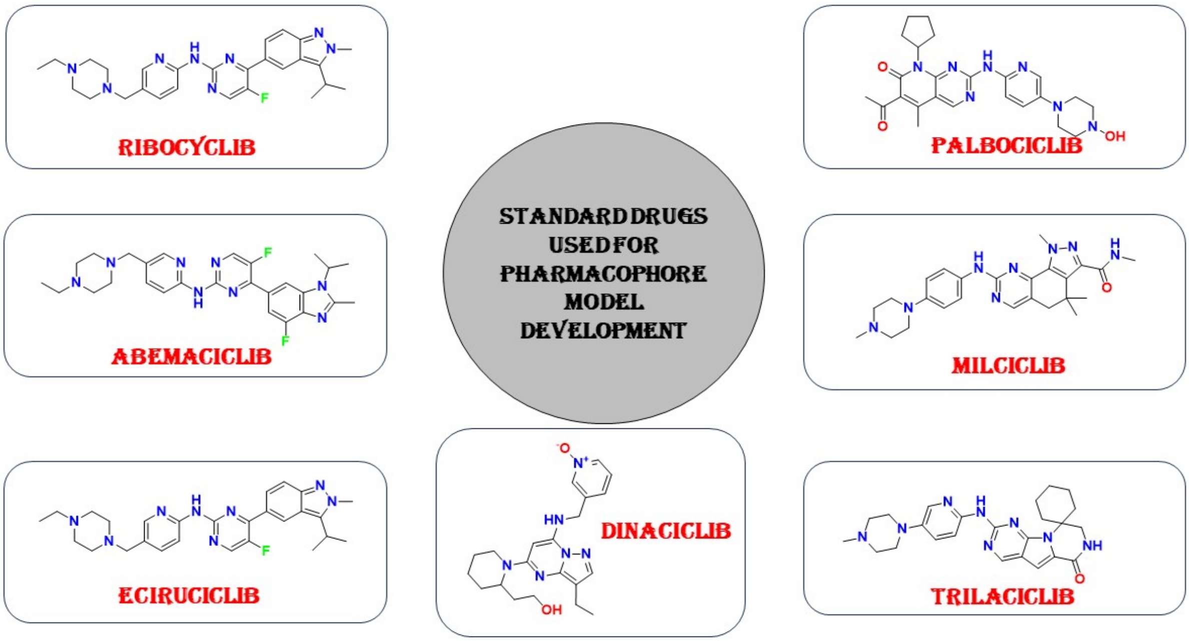

Fig. S1 Approved CDK inhibitor drugs used for Pharmacophore model development.

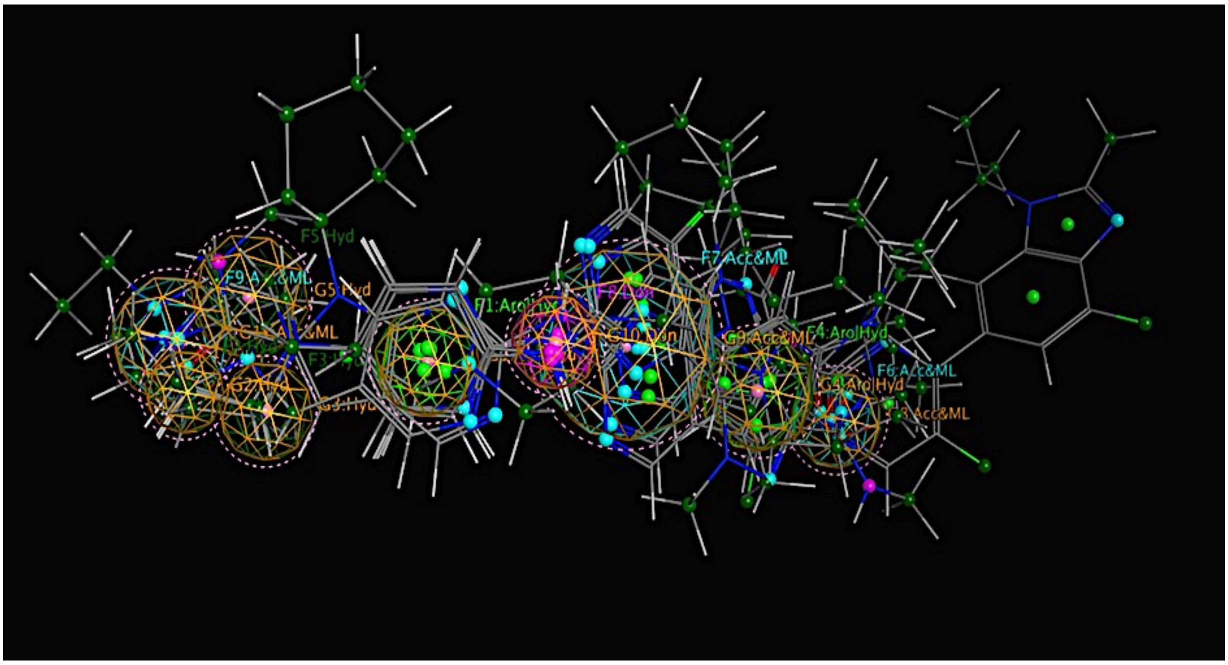

Fig. S2 Pharmacophoric features and Flexible alignment of standard drugs

a) b)

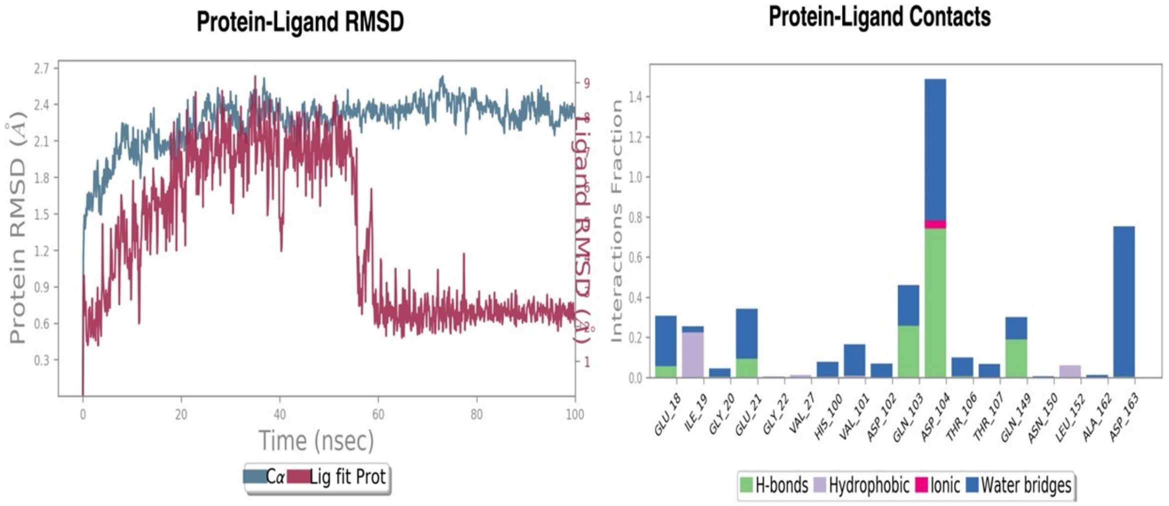

Fig. S3 a) RMSD plot of ligand fit protein of compound 18.

b) H-bond plot of compound 18.

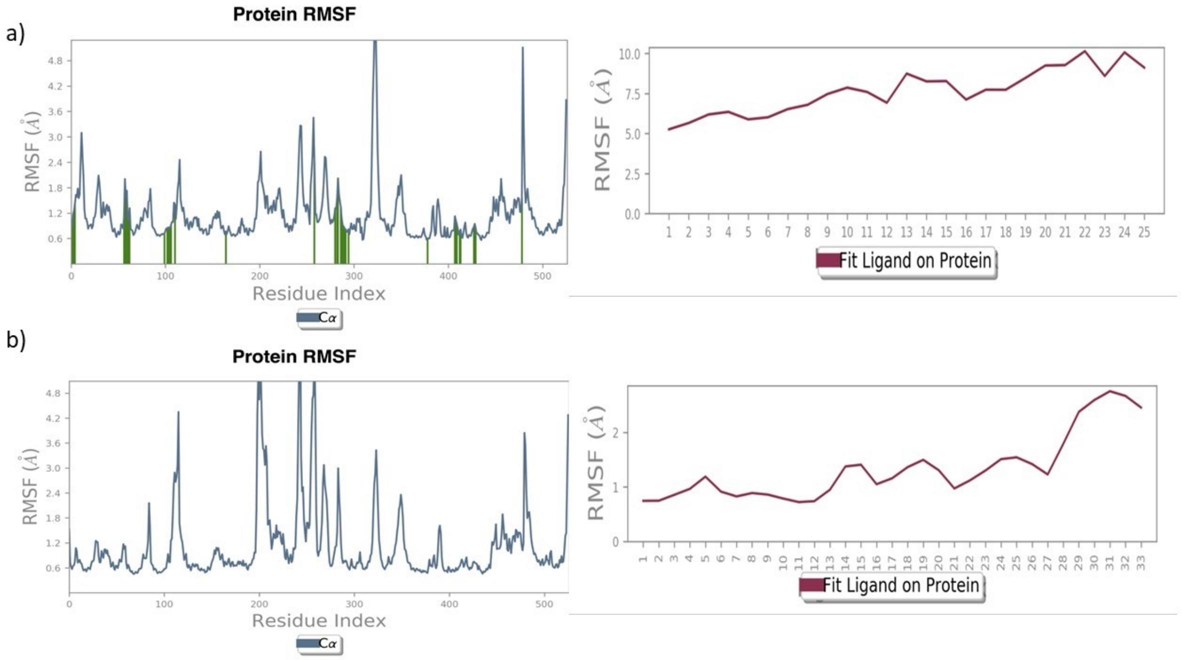

Fig. S4 a) Protein RMSF Reference and Ligand RMSF of Compound-6

b) Protein RMSF Reference and Ligand RMSF of Standard.

Table S1 Physico Chemical parameters of the designed compounds

Ligand ID	log P	TPSA	MW	HBA	HBD	No of rotatable bonds	No of violations
B1	1.54	90.04	314.74	4	2	2	0
B2	1.59	99.27	344.76	5	2	3	0
B3	2.22	90.04	349.18	4	2	2	0
B4	1.50	135.86	359.73	6	2	3	0
B5	1.48	110.27	330.74	5	3	2	0

B6	0.88	119.50	360.76	6	3	3	0
B7	1.08	119.50	360.76	6	3	3	0
B8	0.78	130.50	346.73	6	4	2	0
B9	0.98	130.50	346.73	6	4	2	0
B10	0.51	150.72	362.73	7	5	2	0
B11	1.55	99.27	344.76	5	2	3	0
B12	1.57	99.27	344.76	5	2	3	0
B13	1.36	108.51	374.79	6	2	4	0
B14	1.56	108.51	374.79	6	2	4	0
B15	1.37	117.74	404.81	7	2	5	0
B16	1.17	110.27	404.81	7	2	5	0
B17	1.03	130.50	330.74	5	3	2	0
B18	0.51	150.72	346.73	6	4	2	0
B19	0.28	110.27	362.73	7	5	2	0
B20	1.06	90.04	330.74	5	3	2	0
B21	1.99	90.04	328.76	4	2	2	0
B22	2.32	90.04	393.63	4	2	2	0
B23	2.17	90.04	349.18	4	2	2	0
B24	2.19	90.04	349.18	4	2	2	0
B25	2.80	90.04	383.63	4	2	2	0
B26	3.43	90.04	418.07	4	2	2	0
B27	0.25	102.93	315.72	5	2	2	0
B28	0.37	102.93	315.72	5	2	2	0
B29	0.80	103.18	304.70	5	2	2	0
B30	2.35	90.04	393.63	4	2	2	0

Table S2 Bioactivity score of designed compounds

Ligand ID	GPCR ligand	Ion channel module	Kinase inhibitor	Nuclear receptor ligand	Protease inhibitor	Enzyme inhibitor
**B1**	-0.12	-0.42	0.01	-1.07	-0.62	-0.32
**B2**	-0.14	-0.46	-0.02	-0.97	-0.59	-0.35
**B3**	-0.10	-0.41	0.00	-1.02	-0.61	-0.34
**B4**	-0.25	-0.43	-0.12	-1.02	-0.65	-0.40
**B5**	-0.10	-0.38	0.01	-0.90	-0.58	-0.27
**B6**	-0.11	-0.42	0.02	-0.88	-0.59	-0.28
**B7**	-0.13	-0.39	-0.01	-0.94	-0.61	-0.30
**B8**	-0.07	-0.32	0.03	-0.90	-0.56	-0.24
**B9**	-0.08	-0.36	0.04	-0.78	-0.55	-0.24
**B10**	-0.09	-0.31	0.04	-0.87	-0.53	-0.20
**B11**	-0.15	-0.45	-0.06	-0.97	-0.62	-0.35
**B12**	-0.14	-0.48	-0.04	-0.96	-0.60	-0.35
**B13**	-0.16	-0.44	-0.08	-0.97	-0.60	-0.35
**B14**	-0.13	-0.43	-0.02	-0.89	-0.56	-0.31
**B15**	-0.17	-0.42	-0.07	-0.94	-0.58	-0.33
**B16**	-0.15	-0.42	-0.05	-0.84	-0.57	-0.31
**B17**	-0.06	-0.37	0.05	-0.85	-0.57	-0.26
**B18**	-0.05	-0.35	0.07	-0.79	-0.53	-0.23
**B19**	-0.07	-0.34	0.07	-0.84	-0.50	-0.20
**B20**	-0.05	-0.35	0.07	-0.85	-0.56	-0.25
**B21**	-0.15	-0.48	-0.03	-1.04	-0.63	-0.37
**B22**	-0.22	-0.49	-0.06	-1.15	-0.72	-0.40
**B23**	-0.12	-0.44	-0.11	-1.00	-0.68	-0.39
**B24**	-0.09	-0.40	-0.01	-1.03	-0.61	-0.34
**B25**	-0.09	-0.39	-0.14	-0.95	-0.63	-0.37
**B26**	-0.11	-0.45	-0.21	-0.87	-0.67	-0.35
**B27**	-0.09	-0.37	0.10	-1.09	-0.60	-0.27
**B28**	-0.05	-0.30	0.13	-1.16	-0.58	-0.28
**B29**	-0.31	-0.67	-0.33	-1.33	-1.01	-0.49

**B30**	-0.21	-0.48	-0.03	-1.12	-0.70	-0.39

Table S3 ADMET Studies

**Ligand ID**	**LIVER TOXICITY**		**METABOLISM Cyp inhibitors**						**BBB**	**P-gp inhibitor**	**P-gp substrate**	**Herg Blocker**	**MMP**	**AMES**
	DILI	CYTOTOXIC	HLM	1A2	3A4	2D6	2E9	2C19						
B1	Yes	No	Yes	No	No	No	No	No	Yes	No	No	Yes	No	Yes
B2	Yes	No	Yes	No	No	No	No	No	Yes	Yes	Yes	No	No	Yes
B3	Yes	No	Yes	No	No	No	No	No	Yes	No	No	Yes	No	No
B4	Yes	No	Yes	No	No	No	No	No	No	No	No	No	No	Yes
B5	Yes	No	Yes	No	No	No	No	No	Yes	No	No	No	No	Yes
B6	Yes	No	Yes	No	No	No	No	No	Yes	Yes	No	No	No	No
B7	Yes	No	Yes	No	No	No	No	No	No	No	Yes	No	No	Yes
B8	Yes	No	Yes	No	No	No	No	No	Yes	No	No	No	No	Yes
B9	Yes	No	Yes	No	No	No	No	No	No	No	No	No	No	Yes
B10	Yes	No	Yes	No	No	No	No	No	Yes	No	No	No	No	Yes
B11	Yes	No	Yes	No	No	No	No	No	Yes	Yes	Yes	Yes	No	Yes
B12	Yes	No	No	No	No	No	No	No	Yes	Yes	Yes	Yes	No	Yes
B13	Yes	No	Yes	No	No	No	No	No	No	Yes	Yes	No	No	Yes
B14	Yes	No	Yes	No	No	No	No	No	No	No	Yes	Yes	No	Yes
B15	Yes	No	Yes	No	No	No	No	No	No	Yes	Yes	No	No	Yes
B16	Yes	No	Yes	No	No	No	No	No	Yes	No	Yes	Yes	No	Yes
B17	Yes	No	Yes	No	No	No	No	No	Yes	No	No	Yes	No	Yes
B18	Yes	No	Yes	No	No	No	No	No	Yes	No	No	No	No	Yes
B19	Yes	No	Yes	No	No	No	No	No	Yes	No	No	No	No	Yes
B20	Yes	No	Yes	No	No	No	No	No	No	No	No	No	No	No
B21	Yes	No	Yes	No	No	No	No	No	Yes	Yes	No	No	No	Yes
B22	Yes	No	Yes	No	No	No	No	No	Yes	Yes	No	Yes	No	Yes
B23	Yes	No	Yes	No	No	No	No	No	Yes	Yes	No	Yes	No	Yes
B24	Yes	No	Yes	No	No	No	No	No	Yes	Yes	No	Yes	No	No
B25	Yes	No	Yes	No	No	No	No	No	Yes	Yes	No	No	No	Yes
B26	Yes	No	Yes	No	No	No	No	No	Yes	Yes	No	No	No	No
B27	Yes	No	Yes	No	No	No	No	No	Yes	No	No	Yes	No	Yes
B28	Yes	No	Yes	No	No	No	No	No	Yes	No	No	Yes	No	Yes
B29	Yes	No	Yes	No	No	No	No	No	Yes	No	No	Yes	No	Yes
B30	Yes	No	Yes	No	No	No	No	No	Yes	Yes	No	No	No	Yes
